# Supplementary material for: Hybrid Models and Biological Model Reduction with PyDSTool
Source: PLoS Comput Biol. 2012 Aug 9;8(8):e1002628. doi: 10.1371/journal.pcbi.1002628 (PMC3415397; doi:10.1371/journal.pcbi.1002628)
Supplement: Text S4 — Complete source code for the PyDSTool package (version 0.88.120504). Includes API documentation and help files linking to web pages. This file is identical to the current public release on Sourceforge.net. (ZIP) [file pcbi.1002628.s004.zip › PyDSTool/html/PyDSTool.Variable'.HybridVariable-class.html]

xml version="1.0" encoding="ascii"?


PyDSTool.Variable'.HybridVariable


| Home | Trees | Indices | Help | PyDSTool |
| --- | --- | --- | --- | --- |

Package PyDSTool ::
Module Variable' ::
Class HybridVariable

[hide private]

[frames] | no frames]

# Class HybridVariable

source code

```
object --+    
         |    
  Variable --+
             |
            HybridVariable
```

---

Mimics part of the API of a non-hybrid variable.

This is a somewhat ugly hack as it's implemented by using a whole
HybridTrajectory object to extract individual variable values, rather
than having extracted a sequence of Variable objects from a HT and
stitching them back together as a single entity.


|  |  |
| --- | --- |
| Instance Methods | [hide private] |

|  |  |
| --- | --- |
| \_\_call\_\_(self, indepvar, checklevel=0) | source code |

|  |  |
| --- | --- |
| \_\_del\_\_(self) | source code |

|  |  |
| --- | --- |
| \_\_getstate\_\_(self) | source code |

|  |  |
| --- | --- |
| \_\_init\_\_(self, hybridtraj, coordname, indepdomain, abseps=None)  x.\_\_init\_\_(...) initializes x; see x.\_\_class\_\_.\_\_doc\_\_ for signature | source code |

|  |  |
| --- | --- |
| \_\_repr\_\_(self)  str(x) | source code |

|  |  |
| --- | --- |
| \_\_setstate\_\_(self, state) | source code |

|  |  |
| --- | --- |
| \_\_str\_\_(self)  str(x) | source code |

|  |  |
| --- | --- |
| getDataPoints(self)  Returns a Pointset of independent and dependent variable values, provided variable is based on a mesh (otherwise None is returned). | source code |

|  |  |
| --- | --- |
| info(self, verboselevel=1) | source code |

|  |  |
| --- | --- |
| underlyingMesh(self)  Reveal underlying mesh as arrays, rather than Pointset as returned by getDataPoints method. | source code |

**Inherited from `Variable`**:
`__copy__`,
`__deepcopy__`,
`addMethods`,
`is_continuous_valued`,
`is_discrete_valued`,
`setDepdomain`,
`setIndepdomain`,
`setOutput`,
`truncate_to_idx`

**Inherited from `Variable`** (private):
`_auxfn_getindex`,
`_auxfn_globalindepvar`,
`_auxfn_heav`,
`_auxfn_if`,
`_auxfn_initcond`,
`_infostr`,
`_setRanges`

**Inherited from `object`**:
`__delattr__`,
`__getattribute__`,
`__hash__`,
`__new__`,
`__reduce__`,
`__reduce_ex__`,
`__setattr__`


|  |  |
| --- | --- |
| Properties | [hide private] |

**Inherited from `object`**:
`__class__`


|  |  |
| --- | --- |
| Method Details | [hide private] |

|  |  |
| --- | --- |
| \_\_call\_\_(self, indepvar, checklevel=0)  *(Call operator)* | source code |

Overrides: Variable.\_\_call\_\_

|  |  |
| --- | --- |
| \_\_del\_\_(self)  *(Destructor)* | source code |

Overrides: Variable.\_\_del\_\_

|  |  |
| --- | --- |
| \_\_getstate\_\_(self) | source code |

Overrides: Variable.\_\_getstate\_\_

|  |  |
| --- | --- |
| \_\_init\_\_(self, hybridtraj, coordname, indepdomain, abseps=None)  *(Constructor)* | source code |

x.\_\_init\_\_(...) initializes x; see x.\_\_class\_\_.\_\_doc\_\_ for
signature

Overrides: object.\_\_init\_\_: *(inherited documentation)*

|  |  |
| --- | --- |
| \_\_repr\_\_(self)  *(Representation operator)* | source code |

str(x)

Overrides: object.\_\_repr\_\_: *(inherited documentation)*

|  |  |
| --- | --- |
| \_\_setstate\_\_(self, state) | source code |

Overrides: Variable.\_\_setstate\_\_

|  |  |
| --- | --- |
| \_\_str\_\_(self)  *(Informal representation operator)* | source code |

str(x)

Overrides: object.\_\_str\_\_: *(inherited documentation)*

|  |  |
| --- | --- |
| getDataPoints(self) | source code |

Returns a Pointset of independent and dependent variable values,
provided variable is based on a mesh (otherwise None is returned).

Overrides: Variable.getDataPoints

|  |  |
| --- | --- |
| info(self, verboselevel=1) | source code |

Overrides: Variable.info

|  |  |
| --- | --- |
| underlyingMesh(self) | source code |

Reveal underlying mesh as arrays, rather than Pointset as returned by
getDataPoints method.

Overrides: Variable.underlyingMesh

  


| Home | Trees | Indices | Help | PyDSTool |
| --- | --- | --- | --- | --- |

|  |  |
| --- | --- |
| Generated by Epydoc 3.0.1 on Fri May 4 15:24:10 2012 | http://epydoc.sourceforge.net |
